# Para‐allopatry in hybridizing fire‐bellied toads (*Bombina bombina* and *B. variegata*): Inference from transcriptome‐wide coalescence analyses

**DOI:** 10.1111/evo.12978

**Published:** 2016-07-08

**Authors:** Beate Nürnberger, Konrad Lohse, Anna Fijarczyk, Jacek M. Szymura, Mark L. Blaxter

**Affiliations:** ^1^ Institute of Evolutionary BiologyUniversity of EdinburghAshworth Laboratories, Charlotte Auerbach RoadEdinburghEH9 3FLUnited Kingdom; ^2^ Current Address: Institute of Vertebrate BiologyAcademy of Sciences of the Czech RepublicBrnoCzech Republic; ^3^ Department of Comparative Anatomy, Institute of ZoologyJagiellonian UniversityGronostajowa 9, 30–387KrakówPoland; ^4^ Current Address: Institute of Environmental SciencesJagiellonian UniversityGronostajowa 7, 30–387KrakówPoland

**Keywords:** Ecological speciation, genome‐wide coalescence, hybrid zone, introgression, RNA‐seq

## Abstract

Ancient origins, profound ecological divergence, and extensive hybridization make the fire‐bellied toads *Bombina bombina* and *B. variegata* (Anura: Bombinatoridae) an intriguing test case of ecological speciation. Previous modeling has proposed that the narrow *Bombina* hybrid zones represent strong barriers to neutral introgression. We test this prediction by inferring the rate of gene exchange between pure populations on either side of the intensively studied Kraków transect. We developed a method to extract high confidence sets of orthologous genes from de novo transcriptome assemblies, fitted a range of divergence models to these data and assessed their relative support with analytic likelihood calculations. There was clear evidence for postdivergence gene flow, but, as expected, no perceptible signal of recent introgression via the nearby hybrid zone. The analysis of two additional *Bombina* taxa (*B. v. scabra* and *B. orientalis*) validated our parameter estimates against a larger set of prior expectations. Despite substantial cumulative introgression over millions of years, adaptive divergence of the hybridizing taxa is essentially unaffected by their lack of reproductive isolation. Extended distribution ranges also buffer them against small‐scale environmental perturbations that have been shown to reverse the speciation process in other, more recent ecotypes.

The central role of environmental heterogeneity in driving the evolution of novel ecotypes has been underscored by many recent studies investigating the paradigm of ecological speciation. In particular, there are well‐documented examples of how divergent adaptation can lead to partial reproductive isolation by causing assortative mating within ecotypes, reduced performance of hybrids or both (e.g., Hatfield and Schluter 1999; Rundle et al. 2000; Jiggins et al. 2001; Linn et al. 2003; Ramsey et al. 2003; Huber et al. 2007; see Nosil 2012 for a detailed treatment). Such partial barriers to gene flow can arise even on a historical time scale (Hendry et al. 2007). If this momentum can be maintained then, intriguingly, ecological speciation may run its course relatively swiftly.

And yet, millions of years of divergence are typically required before substantial pre‐ and/or postzygotic isolation is observed in experimental settings (Coyne and Orr 1997; Sasa et al. 1998; Price and Bouvier 2002; Bolnick and Near 2005). Arguably, interbreeding in nature may cease much earlier if divergent adaptation includes novel habitat or mating preferences and/or shifts in the timing of reproduction (Schemske 2010), but barriers to reproduction that are a direct consequence of particular environmental conditions may break down when these conditions change. Spatio‐temporal heterogeneity can work both ways, driving local adaptation and divergence as well as the collapse of intraspecific lineages either through hybridization or extinction (Seehausen et al. 2008). Renewed interbreeding has led to the erosion of differences between recent ecotypes in whitefish (Vonlanthen et al. 2012), sticklebacks (Taylor et al. 2006), and Darwin's finches (Kleindorfer et al. 2014). In the first instance then, young ecotypes have to persist long enough for irreversible reproductive barriers to evolve (Dynesius and Jansson 2014 and references therein). The experiments cited above suggest typical timescales and imply that the process of speciation will often involve shifts in distribution ranges and periods of allopatry (Futuyma 1987; Butlin et al. 2008). Reproductive isolation may then come about by a variety of processes and the relative importance of habitat‐driven adaptive divergence remains to be determined.

Research on ecological speciation has focused mainly on young ecotypes (Faria et al. 2014). This approach helps to establish a clear causal link between adaptive divergence and barriers to gene flow, but necessarily eclipses the question of persistence. Here, we present a case study of two ecologically distinct taxa that have demonstrably persisted for millions of years, yet still interbreed freely wherever their extensive distribution ranges adjoin. We want to know whether the lack of reproductive isolation in any way threatens their continued existence or curtails their independent evolution. Specifically, we estimate the long‐term rate of neutral introgression in populations at a moderate distance from a current hybrid zone and assess the evidence for a recent burst of introgression via this contact zone.

The European fire‐bellied toads, *Bombina bombina* and *B. variegata*, are clearly defined taxa that differ profoundly in a large number of traits, including features of morphology, anatomy, life history, and mating system (see Szymura 1993 for an overview). Many of these differences reflect the distinct ecological niches that the toads occupy. *Bombina bombina* reproduces in semipermanent lowland ponds, whereas *B. variegata* uses ephemeral breeding sites in more mountainous terrain. These aquatic habitats place opposing demands on the tadpoles (Werner and Anholt 1993): cryptic behavior to avoid invertebrate predators versus rapid growth and development as desiccation looms. Developmental, morphological, and behavioral differences between the tadpoles (Rafińska 1991; Vorndran et al. 2002) match established patterns in specialized anuran species along the aquatic permanence gradient (e.g., Relyea 2001). Many differences between the adults are also likely adaptations, for example of *B. variegata* to an ever‐shifting distribution of breeding sites in the landscape and of *B. bombina* to the large mating aggregations that form in ponds. Such simultaneous selection on multiple traits (multifarious selection) may generate particularly efficient barriers to gene flow (Rice and Hostert 1993; Nosil et al. 2009).

*Bombina bombina* and *B. variegata* diverged several million years ago (Szymura 1993; Pabijan et al. 2013) and have experienced repeated cycles of allo‐ and parapatry (Fijarczyk et al. 2011). Present day hybrid zones formed after the last glacial maximum (less than 10 000 years ago; Szymura 1993, 1996). Typically, they are 2–9 km wide and located at the edge of mountainous regions (Fig. 1). In intensively sampled transects in Poland, Hungary, Romania, and Ukraine (Szymura 1976; Szymura and Barton 1986, 1991; Gollmann 1987; Vines et al. 2003; Yanchukov et al. 2006) recombinant genotypes (based on <10 diagnostic loci) predominate, and recombinants make up about a quarter of individuals in the Pešćenica (Croatia) transect (MacCallum et al. 1998). We expect that all individuals in a hybrid zone are recombinant to some degree.

**Figure 1:**
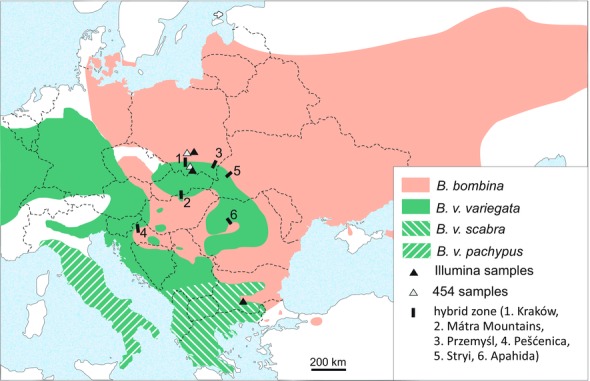
Distribution of *B. bombina* and *B. variegata* in Central and Eastern Europe. Included are the sampling locations (triangles) and the approximate locations of intensively studied hybrid zones. *B. v. pachypus* is included for completeness. This subspecies was not part of the present study. Dashed lines represent national boundaries.

Natural selection counteracts the erosion of differences between the hybridizing taxa. This is shown by the temporal stability of phenotypic clines across 50 and 70 year sampling intervals (Szymura and Barton 1991; Yanchukov et al. 2006) and by the increased mortality of hybrid as opposed to pure embryos under uniform conditions (Szymura and Barton 1986; Kruuk et al. 1999). *Bombina* hybrid zones are proposed to function as partial barriers to neutral gene flow and the barrier strength can be estimated from classic cline theory (Barton and Gale 1993). In particular, the observed significant linkage disequilibria (e.g., Szymura and Barton 1991; MacCallum et al. 1998; Vines et al. 2003) among independently segregating genetic markers (Szymura and Farana 1978; Nürnberger et al. 2003) can be attributed to the influx of pure genotypes into the zone centre. These linkage disequilibria increase the “effective” selection pressure on each selected locus (Barton 1983) such that the associated allele frequency clines become steeper (Slatkin 1973). This triggers a positive feed‐back of even stronger linkage disequilibria and steeper clines and results in a sharp step in allele frequency in the zone center (Barton 1983). It is this stepped pattern, which is observed in all clinal *Bombina* hybrid zones (Szymura and Barton 1991; Yanchukov et al. 2006), which allows us to estimate the genetic barrier to neutral introgression (Nagylaki 1976; Barton 1986).

A robust estimate of barrier strength into the *B. variegata* gene pool comes from transects near Kraków and Przemyśl in Poland where the hybrid zone is ∼6 km wide (Szymura and Barton 1991). Under a diffusion model of dispersal and based on an estimated dispersal range of ∼1 km per generation (Szymura and Barton 1991), this barrier is equivalent to 51 km of unimpeded habitat. Assuming a generation time of three years (Szymura 1988), it would delay the introgression of neutral *B. bombina* alleles by about 8000 years (Barton and Gale 1993). Below, we will compare our new estimates of introgression on either side of the Kraków transect to this expectation.

While genome‐wide linkage disequilibria in the hybrid zone permitted inference from the small number of available diagnostic markers, we need genome‐wide genetic resources to infer the long‐term rate of gene exchange between pure populations. This next step has so far been technically difficult because *Bombina* have “big,” ∼10 Gb genomes (Dufresne and Jeffery 2011; Gregory 2016) without any indication of polyploidy (Olmo et al. 1982). The two sequenced anuran genomes (*Silurana (Xenopus) tropicalis*, Hellsten et al. 2010; *Nanorana parkeri*, Sun et al. 2015) have a most recent common ancestor with *Bombina* 250 Mya, too distant to be useful to design targeted assays. We therefore chose de novo transcriptome assembly as a cost‐effective method to generate reduced representation genomic data (Davey et al. 2011).

Evolutionary or population genomic analyses of next generation sequence data take a locus‐centered view (Cahais et al. 2012) and require the definition of orthologous sequences across samples. With RNA‐seq data, the task of ortholog definition is nontrivial, because variants of a single locus (splice isoforms, alleles) are recovered along with sequences from recent paralogs. The identification of orthologs across samples cannot succeed unless paralogous sequences from each sample are included in the search, but the result is ambiguous if splice isoforms are clustered as well. Similarly, the discovery and genotyping of single nucleotide polymorphisms (SNP) based on read mapping runs the risk of either conflating variation from several loci (false positives) or failing to detect allelic variants across different isoforms (false negatives, De Wit et al. 2015). Moreover, allele‐specific expression is widespread (Chamberlain et al. 2015) and mono‐allelic expression in particular leads to underestimates of genetic variation that can bias downstream inference.

The ortholog problem has been variously addressed in recent de novo transcriptome assemblies. With the help of a reference genome, paralogs can be identified by phylogenetic methods (e.g., Heger and Ponting 2007; Osborne et al. 2013). Alternatively, variant call sets have been screened for suspect genotype distributions in order to remove SNPs that are likely derived from more than one locus (e.g., Gayral et al. 2013). In the absence of a reference genome and starting from either one or two samples per taxon, we devised criteria to identify putative paralogs within transcriptome assemblies and generate a high‐confidence ortholog gene set. Single nucleotide polymorphisms were called by mapping reads to the reference generated from the cognate sample, and pairwise alignments of orthologous reference contigs allowed estimation of within‐ and between‐taxon nucleotide variation. As allele‐specific expression could bias polymorphism estimates, we explored its likely effects on our analysis.

To quantify nuclear divergence and introgression between *B. bombina* and *B. variegata*, we used a recently developed coalescent approach (Lohse et al. 2016) that fits models of divergence with or without gene flow to the data and assesses support via analytically computed likelihoods. This inference is efficient compared to multilocus methods that require computationally intensive Markov chain Monte Carlo sampling (Hey and Nielsen 2004) or simulations (e.g., Shafer et al. 2015). Models were fit by two approaches, one using the site frequency spectrum (SFS) and the other multilocus data summarized by the blockwise SFS, that is the joint occurrence of SFS types in blocks of sequence. The application of this approach to transcriptome data implies discontiguity of blocks and a potential influence of selection on the inference, both of which we consider in detail.

In order to check our inferences against a set of prior expectations, we also estimated divergence and introgression between *B. v. variegata* and two additional *Bombina* taxa, part of a fully resolved phylogeny based on complete mitochondrial genomes (Zheng et al. 2009; Pabijan et al. 2013). *Bombina v. scabra* is a subspecies from the Southern Balkans, and *B. orientalis* (Korea) is the sister taxon of the European lineages and has been geographically separated from them for a long time. We address the following questions: (i) Does the inferred timing and order of divergence between the four *Bombina* lineages from the coalescence analyses match the existing phylogeny? (ii) Is there evidence for postdivergence gene flow between the hybridizing taxa away from the hybrid zone and, if so, how does it compare to the estimate between the *B. variegata* subspecies (genetic barrier to gene flow vs. greater spatial separation)? and (iii) Is there any evidence for a recent burst of introgression as a result of the current hybrid zone? We then relate our results to previous estimates of hybrid zone barrier strength and argue that an extended distribution range is key to the long‐term persistence of the hybridizing taxa and so facilitates the ongoing evolution of reproductive isolation.

## Methods

### SAMPLE ORIGINS

Roche 454 transcriptome sequence data were generated from two females: *B. bombina*, Kozłów, Poland (50º29’N, 20º02’E); *B. v. variegata*, Mogielica, Poland (49º39’N, 20º16’E). Illumina mRNA‐seq data were generated from four individuals: *B. bombina*, male, Sędziejowice, Poland (50°34′N, 20°39′E); *B. v. variegata*, male, Biała Woda, near Szczawnica, Poland (49°24′N, 20°34′E); *B. v. scabra*, female, Rhodopes, Bulgaria; *B. orientalis*, female, Northern clade, Korea. The European sampling locations are indicated in Figure 1. The *B. bombina* and *B. v. variegata* sample sites are on either side of the Kraków transect (Szymura and Barton 1986, 1991) and their distances to the cline center are approximately 61 km (*B. bombina*, Kozłów), 72 km (*B. bombina*, Sędziejowice), 36 km (*B. v. variegata*, Mogielica), and 61 km (*B. v. variegata*, Biała Woda).

### RNA EXTRACTION, LIBRARY PREPARATION, AND SEQUENCING

Liver tissue was used for all RNA extractions. For Roche 454 sequencing, total RNA was extracted using RNAzol®RT, and mRNA was isolated using Dynabeads Oligo(dT)_25_. The RevertAidTM Premium First Strand cDNA Synthesis Kit was used to synthesize the first cDNA strand with a primer complementary to the poly(A) tail with disrupted sequence: 5’‐TTTTTCTTTTTTCTTTTTTV‐3’. The RNA in the RNA/DNA hybrid was cleaved with RNAse H and the second strand synthesized using DNA polymerase I and T4 DNA polymerase. No additional PCR reactions were performed and the cDNA library was not normalized. Two cDNA libraries were sequenced independently (in half an FLX plate each) on a 454 GS FLX Titanium system (454 Life Sciences, Roche) at the Functional Genomics Center in Zürich (Switzerland). For Illumina RNA‐seq, frozen samples in RNA*later*® were thawed and transferred into 1 ml Trizol, homogenized in a TissueLyser and passed five times through a 0.9 mm (20 gauge) needle attached to a syringe (Gayral et al. 2013), followed by 5 min incubation at RT and centrifugation for 1 min at 14,000 rpm. The samples were chloroform extracted once and then mixed with 200 μl of 70% ethanol. Each sample was added to an mRNA spin column (Qiagen Ltd., Manchester, UK) and processed according the manufacturer's instructions. cDNA library preparation and sequencing on an Illumina HiSeq v3 instrument (100 bp, paired‐end) were carried out by Edinburgh Genomics.

### 454 TRANSCRIPTOME ASSEMBLIES

Reads were cleaned by removing residues of poly(A) tails with SnoWhite v.1.1.4 (Barker et al. 2010). Low quality ends were trimmed with PRINSEQ‐lite 0.17 (Schmieder and Edwards 2011). Reads with low‐complexity regions (including stretches of di‐ and trinucleotide repeats that could induce spurious assembly), exact read duplicates and reads that showed significant sequence identity (using BLASTN) to rRNA sequences of *S. tropicalis* were removed. De novo transcriptome assemblies followed a combinatorial strategy (Kumar and Blaxter 2010). Reads were first assembled using Newbler (v2.6, Roche) and Mira v. 3 (Chevreux et al. 2004) and these assemblies were coassembled with CAP3 (Huang and Madan 1999). For Mira and Newbler we used settings recommended for de novo 454 transcriptome assembly, and performed no clipping of poly(A) stretches in MIRA. For CAP3, we used an overlap length cutoff of 80 nucleotides, an overlap identity cutoff of 95%, and switched off the clipping option. To validate the assembly, reads were mapped back to assembled contigs using BLAT (Kent 2002). We retained only contigs with reads that mapped over at least 95% of their length. Eleven percent (*n* = 2729) of *B. bombina* and 14% (*n* = 5490) of *B. variegata* contigs were discarded as a result.

### ILLUMINA TRANSCRIPTOME ASSEMBLIES

Adapters were removed with Cutadapt (Martin 2011) as implemented in Trim Galore! v. 0.3.7 (http:// www.bioinformatics.babraham.ac.uk/projects/trim_galore/), and any sequence downstream of four consecutive bases with mean quality below 25 was clipped with Trimmomatic (Bolger et al. 2014). Sequences with a minimum length of 50 nt were retained (Table 1). De novo assembly was carried out with Trinity (v. 2014‐04‐13p1, Grabherr et al. 2011) in paired‐end mode with the addition of all retained single reads. Default settings were chosen except for the minimum K‐mer count (2), the maximum expected length between fragment pairs (400 nt) and the minimum identity required for the merging of paths during transcript reduction (98%). Nonanuran contaminants were identified by BLAST analysis (megablast) against the NCBI nucleotide database. Contigs with identity >0.90 to Bacteria and Archaea were removed (37–219 contigs per taxon). For each assembly, a BLASTX comparison to the *S. tropicalis* proteome (ftp.xenbase.org/pub/Genomics/JGI/Xentr7.1/Xentr7_2_Stable_Protein.fa.gz, accessed 16 Sept 2014) was carried out with Expect‐value cutoff = 0.01. Over 95% of hits had Expect‐values smaller than 10^−5^. Read support for each contig was quantified with RSEM (Li and Dewey 2011) using a Trinity script. All contigs with fpkm (fragments per kilobase of model per million mapped reads) = 0 were removed from the assemblies (2514–4004 contigs per taxon). Open reading frame (ORF) predictions were computed with TransDecoder from the Trinity packages. Assembly completeness was assessed with CEGMA (Parra et al. 2007).

**Table 1:** RNA‐seq and assembly data summary

	Illumina (Trinity)						Roche 454 (Newbler/Mira/CAP3)			
Taxon	Raw read pairs (x 10^6^)	Read pairs after QC*(x 10^6^)	Single reads after QC (x 10^6^)	Components**	Contigs**	Total assembled bases (x 10^6^)***	Raw reads	Reads after QC	Contigs**	Total assembled bases (x 10^6^)
*B. v. variegata*	55.3	42.3	7.7	66, 454	76, 842	47.4	636, 551	611, 417	27, 650	15.9
*B. v. scabra*	34.4	25.9	5.2	53, 360	60, 406	36.9				
*B. bombina*	47.1	35.9	6.9	60, 768	69, 377	44.5	691, 857	667, 125	21, 127	19.8
*B. orientalis*	46.9	35.3	6.9	51, 811	59, 332	35.7				

*QC = quality control.

**Only components and contigs (min. length ≥ 200 bp) with read support are counted.

***Total assembled bases are computed from the longest contig per component.

### REFERENCE TRANSCRIPTOMES

**Figure 2:**
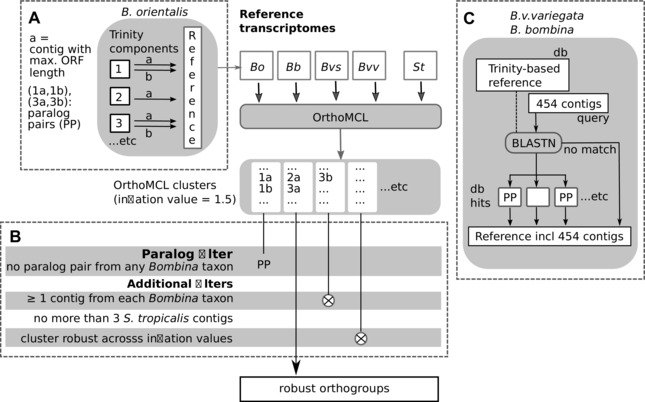
Workflow to generate a set of robust orthogroups. Panel (A) illustrates the definition of the Trinity‐based reference for each *Bombina* taxon. *B. orientalis* serves as an example. Contigs from three components are highlighted and their passage through the pipeline exemplifies the filtering of paralogue pairs (PP). Panel (B) lists the filtering criteria that need to be met by each OrthoMCL cluster in order to be included in the set of robust orthogroups. Panel (C) illustrates the addition of Roche 454 contigs to the Trinity‐based reference of *B. v. variegata* and *B. bombina*. Paralogue pairs were defined as before based on pairwise comparisons of Trinity/Roche 454 contigs in the top ten BLASTN hits. *Bvv* = *B. v. variegata*, *Bvs* = *B. v. scabra*, *Bb* = *B. bombina*, *Bo* = *B. orientalis*, *St* = *S. tropicalis*.

For each taxon, we defined a reference transcriptome that comprised all assembled gene copies (including paralogs) but excluded splice isoforms. Details of the analyses are presented in the Supporting Information A and summarized here (Fig. 2). Trinity components comprise all contigs extracted from a single de Bruijn graph, including isoforms and paralogs. For each component, we identified the contig with the longest ORF (Fig. 2A) and analyzed pairwise alignments of all other contigs with it. In the first instance, all contig pairs with overall sequence identity below 0.98 were designated as paralogs. In the case of markedly heterogeneous identity scores along the alignment, we distinguished poorly aligned tracts due to structural variation near alignment termini from those due to the alternate use of duplicated exons. The former tracts were excluded from identity computations. The latter case represents paralogy below the level of the gene (*cf*. Gabaldón and Koonin 2013) and contig pairs were designated accordingly, even if the mean identity was greater or equal 0.98. For *B. bombina* and *B. v. variegata* Roche 454 assemblies, we used BLASTN to determine paralog status in comparison to the Trinity assemblies within high scoring pairs as before (Fig. 2C). For each taxon, the reference consisted of one contig per Trinity component (with maximum ORF length), all associated paralogs and, if present, all Roche 454 contigs (with or without paralog status).

### ORTHOLOG IDENTIFICATION AND ALIGNMENT

We used OrthoMCL (Li et al. 2003) with default parameters to cluster predicted peptide sequences from all four reference transcriptomes (Fig. 2). The complete proteome of *S. tropicalis* was added as a “backbone” for clustering and to provide additional information on multigene families. From the initial clustering (inflation value = 1.5), we retained only those groups that comprised the following (Fig. 2B): (1) no paralog pairs from any *Bombina* taxon, (2) at least one contig from each *Bombina* taxon and (3) no more than three *S. tropicalis* contigs. From this subset of clusters we kept only those that were robust to changes in the inflation value (range: 0.5–5.0) in order to counter common errors of graph‐based methods such as OrthoMCL, that is overclustering and incorrect handling of domain recombination (Kristensen et al. 2011). For these robust orthogroups, peptide alignments were computed between pairs of *Bombina* taxa. Note that we applied stringent criteria to obtain a set of robust orthogroups rather than an accurate list of paralogs. False positives in the paralog list were therefore of no concern.

### VARIANT DETECTION

For the coalescence analyses, only the Illumina datasets were considered (corresponding to one diploid individual per taxon). For each taxon, reads were mapped to the reference transcriptome (for *B. bombina* and *B. v. variegata*: Trinity contigs only) with Bowtie2 v.2.2.4 (Langmead and Salzberg 2012, mode = sensitive). Variant detection was carried out with GATK (McKenna et al. 2010; de Pristo et al. 2011): duplicates were removed, isoforms were split as required (Split'N'Trim) and base qualities were recalibrated once to convergence. Raw variants were called with the HaplotypeCaller (genotyping mode = Discovery, stand_emit_conf = 10, stand_call_conf = 20) and filtered following GATK recommendations for RNA‐seq data (see Supporting Information A). For a given taxon pair, peptide alignments within robust orthogroups were back‐translated into the nucleotide sequences of the reference contigs with PAL2NAL (Suyama et al. 2006). For a given orthogroup and taxon, an alternate haplotype was constructed from the variant call format (VCF) file and added to the between‐taxon alignments. These were parsed for variation at fourfold degenerate sites.

### COALESCENT ANALYSES

We used two complementary strategies to estimate models of divergence with and without gene flow for each pairwise comparison of *B. v. variegata* against increasingly diverged taxa (*B. v. scabra, B. bombina, B. orientalis)*. For an unrooted alignment of a pair of diploid samples, there are four types of sites: (i) heterozygote in taxon A (k_A_), (ii) heterozygote in taxon B (k_B_), (iii) heterozygote in both A and B (k_AB_), and (iv) alternate homozygotes (k_AABB_). Counts of these four site types, *k* = {*k*_A_, *k*_B_, *k*_AB_, *k*_AABB_}, were obtained from blocks of 150 consecutive fourfold degenerate sites. First, we based inference on the average frequencies of these site types *E[k*_i_], using one randomly picked variable site per block. This yields essentially the folded, joint site frequency spectrum (SFS) for two diploid samples. Second, we used our observations of the joint distribution of the four site types (*k*) to fit models of divergence and gene flow. We will refer to this as the blockwise SFS (bSFS) throughout. Assuming blocks are unlinked, the overall model likelihood is given as the product of *p(*k*)*, the probability of each observed blockwise configuration of mutations across blocks.

These two approaches trade power against bias. The SFS ignores all linkage information and summarizes the data into four site frequencies. In principle, this allows us to coestimate at most four parameters. However, the near absence of shared heterozygote sites, E[*k*_AB_], restricted our analyses to three‐parameter models. This limitation is offset by more robust inference. In particular, point estimates based on the SFS are not affected by recombination within blocks or heterogeneity in mutation rate. In contrast, the analyses based on the bSFS assume a constant mutation rate across blocks and no recombination within them. Violations of these assumptions produce biased estimates. This is of particular concern for transcriptome data, given the added potential for recombination in introns. However, by considering the joint distribution of linked mutations, the bSFS allows us to fit more complex models.

**Table 2:** Heterozygous single nucleotide polymorphisms determined by GATK

	Total assembly				SNPs in the coalescence data set			
Taxon	# SNPs	% SNP (GQ**= 99)	Major allele frequency*	Median read coverage	# SNPs	% SNPs (GQ**= 99)	Major allele frequency*	Median read coverage
*B. v. variegata*	79, 349	53.6	0.6670	15	407	90.9	0.6068	33
*B. v. scabra*	80, 910	54.2	0.6571	15	567	81.3	0.6099	25
*B. bombina*	64, 471	48.4	0.6718	13	240	85.0	0.6132	30
*B. orientalis*	122, 445	59.8	0.6397	16	1346	81.2	0.6087	24

*The major allele frequency is computed for reads from a single individual that cover a given SNP position.

**GQ = phred‐scaled genotype quality.

**Figure 3:**
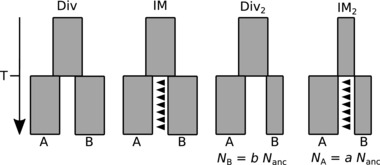
Sketches of the four coalescence models. The block widths indicate relative effective population sizes in the ancestral lineage and its two descendants A and B. Arrows mark the direction of gene flow. For a given model, we depict the gene flow direction and/or relative population sizes that gave the best fit for *B. v. variegata* (A)*/B. bombina* (B). Note that all possible combinations were evaluated for each model and taxon pair. Divergence time *T* is estimated in units of 2*N*_ANC_.

Lohse et al. (2016) have recently used a recursion for the generating function of genealogies (Lohse et al. 2011) to derive expressions for both *E[k*_i_] and *p(*k*)* under a model of isolation with unidirectional migration (IM). The model assumes an ancestral population of constant effective size *N*_anc_ that splits into two populations A and B at time *T* (measured in 2*N*_anc_ generations), followed by unidirectional migration at a constant rate of *M = 4N*_anc_ *m* individuals per generation, where *m* is the proportion of immigrants per generation. Note that it is currently not possible to fit a more general model of bidirectional gene flow. We refer to the null model without migration as Div (Fig. 3).

Asymmetry in the frequency of unique heterozygous sites in each species (k_A_ and k_B_) may be due to differences in effective population size, asymmetry in postdivergence gene flow or both. We therefore also considered a model in which the *N*_e_ in one of the descendant species was allowed to change instantaneously after divergence, while the N_e_ of the other species remained constant (Fig. 3). We contrasted the relative support for this model without gene flow (Div_2_, *M* = 0) with that for its counterpart, IM, with gene flow but a single N_e_ for all three populations. These models with three parameters were fitted using the SFS and the bSFS. Finally, we used the bSFS to fit models that allow for both postdivergence gene flow and asymmetries in N_e_ (IM_2_). In each case, we tested for gene flow and asymmetries in N_e_ in either direction (i.e., *N*_A_ = a *N*_anc_ or N_B_ = b *N*_anc_ and *M*_AB_ or *M*_BA_) which four possible models. If gene flow is bidirectional and asymmetric, the best‐supported model should identify the majority direction.

We assume that blocks are randomly distributed and, given the large size of the *Bombina* genome (2600 cM, Morescalchi 1965), that the effects of physical linkage between them can be ignored (a rough calculation gives a maximum density of one block per 1.75 cM for *B.bombina/B.v.variegata*). Given the total tree‐length, this distance implies thousands of recombination events. We therefore treat each block as an independent replicate of the coalescent. For the SFS, we obtained 1000 bootstrap replicates of one randomly drawn variable site per block and estimated 95% confidence intervals of parameter estimates as 1.96 SD of parameter estimates across these replicates. The dataset comprised more than 1000 blocks with 150 fourfold degenerate sites and an average of 2.7–6.0 variable sites per block (depending on the comparison). Longer blocks would have contained more variable sites each, but they were much less numerous in the dataset (preliminary analyses, not shown) and more likely to produce biases due to within‐block recombination. Maximum likelihood estimates were obtained using the *Nmaximise* function in Mathematica (v. 10.2, Wolfram Research, Inc., Champaign, IL, USA, for details see Lohse et al. 2016 and the Mathematica notebook, Supporting Information B).

### ESTIMATION OF SYNONYMOUS SUBSTITUTION RATES

In order to convert coalescence time estimates, *T*, into absolute times, *t*, we derived an estimate of the substitution rate per generation, μ, at fourfold degenerate sites from a recent anuran phylogeny based on nine nuclear genes (*rag1, rag2, bdnf, pomc, cxcr4, slc8a1, scl8a3, rho, h3a*, Irisarri et al. 2012b). That study demonstrated considerable rate variation between Neobatrachians and Archaeobatrachians (including *Bombina*). We therefore used data from two taxa in the latter group, *Alytes* and *Discoglossus* (MRCA 153 Mya, Roelants et al. 2011), which belong to the sister family of the Bombinatoridae. From the concatenated nuclear dataset (Irisarri et al. 2012a), 896 fourfold degenerate sites were extracted and the substitution rate per year was estimated with PAML v. 4.7 (Yang 2007). Given the three year generation time of *Bombina*, we obtained μ = 9.15×10^–9^.

## Results

### NEW TRANSCRIPTOMES FOR *BOMBINA* SPECIES

We generated new transcriptome data and assemblies for six individual toads from four *Bombina* species and subspecies using Roche 454 and Illumina technologies. The assemblies were of high individual quality. Among the components generated by the Trinity assemblies (Table 1), 38–46% were annotated with an ORF prediction and/or a BLASTX hit to the *S. tropicalis* transcriptome (expect‐value cut‐off 1×10^–5^). The total number of annotated components ranged from 24,204 (*B. orientalis*) to 26,038 (*B. v. variegata*). Over 90% of 248 ultraconserved core eukaryote genes (CEGs) were either partially or completely (>70% of total protein length) assembled for each taxon. The contigs that lacked annotation were relatively shorter and had lower coverage. For example in *B. v. variegata*, contigs without ORF had a median length of 411 bp compared to 1214 bp for contigs with ORF. The analogous figures for coverage were 1.14 versus 2.19 (median fkpm) and 3.76 versus 27.3 (mean fpkm), respectively. Similarly in the Roche 454 assembly, *B. v. variegata* contigs without ORF (*n* = 19,088) had a median length of 458 bp compared to 700 bp in contigs with ORF (*n* = 9739). Because all downstream analyses were based on coding sequence, only the assembly subsets with ORF predictions are considered below.

### PARALOG IDENTIFICATION

In the Trinity assemblies, 16–20% of components with ORF contained more than one contig and were screened for paralogs (2791–3715 components per taxon). In *B. v. variegata* for example, 10,753 screened contigs contained 3715 contigs with greatest ORF length (“a” contigs in Fig. 2A) and 1767 paralogs (“b” contigs). The remaining 5271 contigs were not further analyzed as they presumably contained alleles and isoforms. For the other taxa, the paralog counts were 1375 (*B. v. scabra*), 1737 (*B. bombina*), and 1422 (*B. orientalis*). Among the Roche 454 contigs with ORFs, 9172, and 7866 had a BLASTN match to a Trinity contig in *B. v. variegata* and *B. bombina*, respectively (Fig. 2C). Of these, 3375 (*B. v. variegata*) and 2626 (*B. bombina*) were identified as paralogs. This relatively greater number of paralogs in the 454 assemblies reflects the overall lower identities in the BLAST‐derived sequence pairs compared to those within Trinity components. Note that only the most similar Trinity/Roche 454 paralog pairs will affect the definition of robust orthogroups (Fig. 2B).

### ORTHOLOG IDENTIFICATION

Of the 15,775 OrthoMCL clusters with more than one *Bombina* taxon, 1719 were excluded because they comprised at least one pair of paralogous contigs. There were 6772 groups from which at least one of the four *Bombina* taxa was missing. An excess (>3) of *S. tropicalis* contigs excluded 3845 groups. Many clusters failed to meet more than one filtering criterion. *Bombina* paralogs were the sole reason for exclusion in 716 cases. After these filtering steps, 4978 clusters remained. Among these, 4040 were robust to changes in the inflation value.

The inclusion of presumptive paralogs in the clustering analysis not only led to the removal of groups with unresolved orthology (i.e., those with paralog pairs) but also allowed for a sequence‐based decision which member of a paralog pair to include in a given group (see Supporting Information A for further details and an example).

### VARIANT DETECTION

Just under 10% of the variants reported by GATK for each taxon were indels and removed from further analysis. We observed a small number of genotype calls (2861–4678 per taxon) that did not include the reference allele (i.e., alternate homozygotes). Because references (Trinity‐based) and mapped reads (Illumina) came from a single individual per taxon, these largely low quality genotypes (Phred score <20 in 75% of cases) are likely to represent assembly errors and were removed. Genotype qualities greater than 40 were assigned to over 90% of the remaining heterozygous SNPs (Table 2) and to all but two SNPs in the coalescence dataset. In this subset, more than 80% of SNPs reached the maximum reported quality of 99 (Table 2) and no alternate homozygotes were observed.

Our ability to discover SNPs, especially in the case of allele‐specific expression, depends on read depth. An upper bound for this is the mean read coverage (range: 140–200 fold per taxon), which was computed after quality control from the total assembly length, the read counts (Table 1) and the mean read length. PCR duplicates inflate these figures and GATK's “deduping” algorithm reduced each read set by about 80%. The resulting median read depth (Table 2) is an underestimate, because high coverage sequencing of a relatively short template will have produced independently primed read pairs that are identical to each other. Variation in read depth did not drive the striking taxon differences in levels of variation. SNPs in the *B. orientalis* coalescence dataset (lowest median read depth, Table 2) outnumbered those in *B. v. variegata* (highest median read depth) almost fourfold. Thus, while allele‐specific expression featured in this dataset (Supporting Information A), our inferences are robust to its effects (see Discussion).

For the three pairwise comparisons, the number of robust orthogroups that contained a block of 150 fourfold degenerate sites ranged from 1179 (*B. v. variegata* vs. *B. orientalis*) to 1479 (*B. v variegata* vs. *B. bombina*, Table 3). At fourfold degenerate sites, the average heterozygosity (k_A_, k_B_) ranged from 0.1% in *B. bombina* to 0.7% in *B. orientalis* (Table 3). As predicted by the mitochondrial phylogeny (Pabijan et al. 2013), the average frequency of homozygous differences (k_AABB_) was lowest between the *B. variegata* subspecies, intermediate between *B. variegata* and *B. bombina* and greatest between *B. variegata* and *B. orientali*s (Table 3).

**Table 3:** Average per site frequencies of the four mutation types for each pairwise comparison of *B. v. variegata* (Taxon A) versus another taxon (Taxon B)

Taxon B	Method	Source	k_A_	k_B_	k_AB_	k_AABB_	Number of ortho‐blocks*
*B. v. scabra*	SFS	obs	0.00178	0.00275	0.0000444	0.0136	1352
	bSFS	obs	0.00174	0.00274	0.0000198	0.0136	1347
		exp**	0.00168	0.00266	0.0000188	0.0134	
*B. bombina*	SFS	obs	0.00181	0.00106	0.0000180	0.0187	1481
	bSFS	obs	0.00177	0.00106	0.0000090	0.0187	1479
		exp	0.00155	0.00119	0.0000039	0.0185	
*B. orientalis*	SFS	obs	0.00172	0.00748	0.0000616	0.0308	1190
	bSFS	obs	0.00162	0.00744	0	0.0308	1179
		exp	0.00154	0.00752	0	0.0312	

*The number of orthoblocks differs slightly between analyses, because all blocks that violated the four gamete test were excluded from the bSFS.

**Expected values from the best fitting model (Table 4, bold figure)

### COALESCENCE ANALYSES

In both parapatric taxon pairs (*B. v. variegata/B. v scabra* and *B. v. variegata/B. bombina*), models with postdivergence gene flow fit the data significantly better than those without (Table 4). This was true regardless of whether we allowed for differences in *N_e_* or not, or whether the inference was based on the SFS or the bSFS. Estimates of gene flow (*M*) were low (<0.1) in both comparisons, but *M* estimates (both based on the SFS and the bSFS, Table 5) were significantly greater between the *B. variegata* subspecies than between *B. v. variegata* and *B. bombina*.

**Table 4:** Model support, measured as the difference in ln(Likelihood) relative to the best model (zero values, bold)

Taxon B*	Method	Model** Div	IM_A→B_	IM_B→A_	Div_2_	IM_2,A→B_	IM_2,B→A_
*B. v. scabra*	SFS	−30.2	**0**	−13.2	−22.4		
	bSFS	−60.0	−26.1	−31.1	−39.3	−25.6	**0**
*B. bombina*	SFS	−14.4	−4.24	**0**	−11.7		
	bSFS	−62.5	−30.7	−4.8	−48.1	−7.5	**0**
*B. orientalis*	SFS	−64.7	**0**	−55.8	−14.7		
	bSFS	−223	−223	−223	**0**	Div_2_ †	Div_2_ †

*Comparisons to *B. v. variegata* (Taxon A), as in Table 3.

**Div = strict divergence; fixed *N*_e_, IM = divergence with migration, fixed *N*_e_; Div_2_ = strict divergence, asymmetric *N*_e_; IM_2_ = divergence with migration, asymmetric *N*_e_. Migration is unidirectional (A→B or B→A). For Div_2_ and the two IM_2_ models, both *N*_e_ asymmetries were evaluated (*N_A_* = *a N*_anc_ and *N_B_* = *b N*_anc_). We report the best fit in each case (see also Figure 3, Table 5).

†The IM_2_ models collapsed to a strict divergence model (Div_2_).

**Table 5:** Maximum likelihood estimates of parameters under the best supported model (Table 4)

Taxon B*	Method	θ	*M*	*T* **	*N*_e_ factor***
*B. v. scabra*	SFS	0.00179	0.078 (0.055, 0.099)	12.5 (9.2, 15.7)	n/a
	bSFS	0.00129	0.032 (B→A)	12.0	2.07 (B)
*B. bombina*	SFS	0.00107	0.027 (0.013, 0.041)	22.4 (16.6, 28.1)	n/a
	bSFS	0.00154	0.015 (B→A)	19.8	1.36 (A)
*B. orientalis*	SFS	0.00277	0.136 (0.120, 0.150)	∞	n/a
	bSFS	0.00752	0	3.75	0.2 (A)

*Comparisons to *B. v. variegata* (Taxon A), as in Table 3.

**The divergence time (*T*) is given in units of 2 *N_anc_* generations (95% CI in brackets).

***N_e_ factor (*a* or *b* in Figure 3) multiplied with *N*_anc_ equals *N*_e_ for one of the descendant populations (in parentheses).

n/a = not applicable.

Our estimates of the divergence time also agreed well between SFS and bSFS analyses (Table 5). We converted estimates of *T* (Table 5) into absolute time using *t = T* × *2N* × *g*, where *g* is generation time and *N* = θ/(4μ), using an estimate of μ (9.15×10^–9^) from a related pair of Archaeobatrachians. In the bSFS analyses, this gave ancestral population sizes of 0.35 – 2×10^5^. The divergence times for *B. v. variegata* and *B. v. scabra* were 3.6 Mya (SFS) and 2.5 Mya (bSFS). For the other comparisons, divergence times were 3.9 and 3.3 Mya (*B. v. variegata/B. bombina*) and 4.6 Mya (*B. v. variegata/B. orientalis*).

The SFS and bSFS analyses inferred different population histories for the allopatric pair (*B. v. variegata/B. orientalis*, Tables 4 and 5). Using the SFS, the IM model with a single *N_e_* resulted in estimates of comparatively high gene flow (*M* = 0.136) from *B. v. variegata* to *B. orientalis* and an infinite divergence time. This was most likely an artefact of the large *N_e_* of *B. orientalis*. Using the bSFS, a model of strict divergence with varying *N_e_* (Div_2_) gave a better fit than a simple IM model and allowing for both gene flow and varying *N_e_* (IM_2_) did not improve model fit. In the case of the two *B. variegata* subspecies, the bSFS analyses gave significantly better support for gene flow in the opposite direction (i.e., from *B. v. scabra* to *B. v. variegata*). Finally, estimates of *M* were lower in the bSFS than the SFS analyses (Table 5).

**Figure 4:**
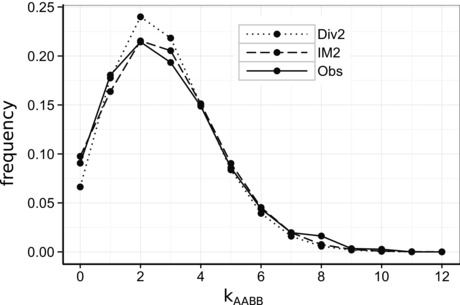
The distribution of homozygous differences between *B. v. variegata* and *B. bombina* (k_AABB_) in blocks of 150 fourfold degenerate sites. The divergence model predicts a narrower distribution (dotted lines) than observed (solid lines), that is we observe an excess both of blocks with no fixed differences and of blocks with a large number of fixed differences (*k*_AABB_ > 6). An IM_2_ model with migration and asymmetry in N_e_ (dashed line) gives a good fit to the data.

We checked whether the rare cases of shared heterozygous sites had undue leverage on our inference. For *B. variegata* and *B. bombina*, only one block contained such sites and removing it did not alter the gene flow signal. Instead, most of this signal came from an excess of blocks with no fixed differences (k_AABB_ = 0, Fig. 4). For *B. variegata* and *B. bombina* there are 134 such blocks (total: 1479) which is highly unlikely under the best fitting divergence scenario (*P* = 0.002 assuming binomial sampling), but not under the IM_2_ model (*P* = 0.83). The earliest signal of introgression via the nearby hybrid zone would be generated by uniformly adaptive variants that would produce an excess of invariant blocks (k = {0,0,0,0}). However using analogous coalescent calculations, we find that the observed number of such blocks (95) is not unexpected under the IM_2_ model (*P* = 0.149). To assess the absolute fit of the best‐supported models, we compared the observed SFS to those expected under the bSFS‐based models and found a good overall match (Table 3). This suggests that there would be insufficient information in the data to evaluate more complex models (e.g., with variable migration rates).

## Discussion

Our coalescent analyses show that despite their ancient divergence, *B. bombina* and *B. v. variegata* have continued to exchange genes, at a low rate (*M*_BA_ = 0.015 or 0.027, depending on the analysis). Thus despite the abundance of fertile hybrids in contact zones, this effective rate of gene exchange between the two pure populations corresponds to no more than one immigrant per 110–200 years. These values are almost 100 times smaller than an estimate by the same method for two sympatric *Heliconius* species that hybridize infrequently (Lohse et al. 2016). However, given the old divergence time (on the coalescent timescale), the *Bombina* figures imply that a substantial portion of genes has been affected by past introgression: based on the results from the bSFS analysis, this proportion for *B. v. variegata* is 1 – e^–0.015 × 19.8^ = 0.257 (Table 5). The IM_2_ model of gene flow in the opposite, less well‐supported direction gave an estimate of *M*_AB_ = 0.0127. While postdivergence gene flow has likely been bidirectional, the stronger support for introgression into the *B. variegata* gene pool agrees with observations from current hybrid zones. A much wider spectrum of hybrid genotypes is found in puddles ( = *B. variegata* breeding sites) than in ponds (e.g., MacCallum et al. 1998, Vines et al. 2003). Before we explore our results in more detail, we comment on our methodology and check our results against prior expectations.

Our use of RNA‐seq data for evolutionary inference without a reference genome required careful specification of an ortholog gene set. By identifying putative paralog pairs within each of four assemblies and then including these in the cross‐taxon clustering step, we could eliminate clusters with unresolved orthology and improve orthology assignments overall. Several additional filtering steps ensured that the coalescence analyses were based mainly on relatively long, commonly expressed genes with high‐confidence orthologs across all four *Bombina* taxa. Variant detection in this subset will have been largely based on uniquely mapping reads and has produced a set of high quality SNPs. However, some errors will remain, in particular due to undetected differential gene loss (Rasmussen and Kellis 2012).

SNPs have no doubt been missed due to allele specific expression and/or low read depths for particular genes. However as we argue above, the reported median read depths (Table 2) are underestimates resulting from stringent duplicate removal by GATK. True mitotically stable monoallelic expression (<5% of genes per cell type in *Mus*, Reinius and Sandberg 2015) contributes to underestimates of genetic variation and of shared heterozygosity in particular. Consequently, our inference of low but significant gene flow between the hybridizing taxa is conservative.

Interpretation of the coalescent results is conditioned by the strengths and weaknesses of thetwo inference schemes. The bSFS assumes no recombination within blocks. Our blocks of 150 fourfold degenerate sites were on average 1500 bp long. In the two fully sequenced anuran genomes, the average CDS length of 1323 bp (*S. tropicalis)* and 1382 bp (*N. parkeri*) corresponded to average gene lengths of 18.0 and 24.4 kb, respectively (Sun et al. 2015). This suggests that the genomic complement of each block corresponds to an average span of >20 kb. Recombination within blocks is thus likely to influence our parameter estimate. It should reduce the variance in coalescence times and lead to lower estimates of *M* and *N*_e_ (Wall 2003) compared to the SFS analyses, which was indeed the case (Table 5). However the biases appear surprisingly small and for a given model the results from both approaches agreed well. Selective sweeps at linked sites in the ancestral population may have the opposite effect of inflating the variation in coalescence times (Coop and Ralph 2012). The excess of blocks with no or few divergent sites in the European comparisons may in principle be explained in this way. However, the fact that no such excess is seen in the allopatric comparison (*B. v. variegata/B. orientalis*) suggests that it is instead a genuine signal of past migration.

Use of the SFS to fit models requires fewer assumptions, but this approach has little power to distinguish gene flow from differences in effective population size since the lineage split. Thus, when assuming a single *N*_e_, we always inferred gene flow into the taxon with higher heterozygosity and obtained unrealistic estimates for the allopatric taxon pair (Table 5). The bSFS contains additional information that helps to resolve the ambiguity: a larger *N*_e_ in one descendant population increases heterozygosity uniformly whereas gene flow generates a small number of blocks with large numbers of heterozygous sites in the population receiving migrants. Thus allowing for two *N*_e_ parameters in the bSFS analyses, *M* estimates were reduced to zero as expected (*B. v. variegata/B. orientalis*) or the direction of gene flow was reversed (*B. v. variegata/B. v. scabra*).

The three pairwise taxon comparisons allow us to assess the robustness of our inference. The ranking of observed heterozygosities (Table 3) agrees with previous genetic analyses and inferred Pleistocene phylogeography (Szymura 1988, 1993; Fijarczyk et al. 2011). For example, genetic diversity was lowest in *B. bombina* (k_B_ = 0.00106), which recently expanded its range from glacial refugia near the Black Sea, and greatest in *B. orientalis* (k_B_ = 0.00748) from Korea, an important Pleistocene refugium for Asian taxa (Zheng et al. 2009). Moreover, the inferred direction of migration between the two *B. variegata* subspecies (IM_2,B→A_) matches previous findings of relatively more gene flow from the Balkans into the Carpathians (Fijarczyk et al. 2011).

The ranking of species divergence times also agrees with the established phylogeny of the taxa, but the estimates are more recent than those inferred from mitochondrial DNA. For example for the split between *B. v. variegata* and *B. bombina*, we obtain 3.3 Mya (bSFS) compared to the 6.4 or 8.9 Mya divergence at mtDNA genes (two different fossil calibrations, Pabijan et al. 2013; see also Heled and Drummond 2015). While the coalescence time of any particular gene should indeed predate the lineage split, the magnitude of the difference is surprising and likely reflects differences in calibration. The new estimate is consistent with the appearance of the first *B. bombina* fossils in the late Pliocene (Rage and Roček 2003) and with analyses of allozymes (Nei's D, Szymura 1993), but uncertainties clearly remain. We therefore conclude cautiously that *B. bombina* and *B. v. variegata* diverged in the mid‐Pliocene, at a time when much of their present‐day distribution range in Central Europe was still covered by remnants of the Paratethys Sea. Subsequent climatic oscillations and associated shifts in distribution ranges presumably provided recurring opportunities for gene exchange that we detect in present‐day transcriptomes.

Is there evidence for a burst of recent introgression *via* the nearby hybrid zone? Neutral mixing should erode differentiation and so lead to an excess of blocks without homozygous differences (k_AABB_ = 0). Such blocks were indeed overrepresented under a model of strict divergence, but their number was compatible with a single long‐term migration rate. Selectively favored alleles should introgress much more quickly (Barton 1979). For example, an allele with a 1% selective advantage is expected to traverse the Kraków transect in a mere 245 years (via expression in Barton and Gale 1993, p. 30 top). Such a wave of advance leaves invariant blocks (*k* = {0,0,0,0}) in its wake, but the observed frequency of invariant blocks was not higher than expected under a model of long‐term neutral gene flow. While this does not rule out adaptive introgression at some of the genes in our dataset, larger samples of individuals and long‐range linkage information would be needed to detect these loci.

The lack of a migration signal from the current hybrid zone agrees with the predictions from cline theory (see Introduction): given the barrier effect (51 km) and the distance of the *B. v. variegata* sample from the hybrid zone (61 km), it should take roughly 18,500 years for neutral variants to cross the Kraków transect and arrive at Biała Woda. Assuming similar barrier strengths, the *B. bombina* sample (72 km) should be insulated even longer from introgression *via* that route. The time scale of introgression will be even longer for those SNPs closely linked to variants that are at a selective disadvantage once they have crossed the hybrid zone. Our estimate of the long‐term effective number of migrants, *M*, implies a rate of gene exchange, *m*, on the order of 10^–6^.Thus a few dozen dispersal ranges from the hybrid zone, adaptive divergence proceeds essentially unaffected by the lack of reproductive isolation between the taxa. Similarly low estimates of *m* were obtained for hybridizing sunflowers (Sambatti et al. 2012).

The strength of the genetic barrier is much weaker in the *Bombina* contact zone near Apahida, Romania. There, the two taxa meet in an extended habitat mosaic of puddles and ponds at a distance of 20 km from the nearest pure species range (Vines et al. 2003). Even if we assume the most efficient genetic barrier, that is hundreds of selected loci evenly distributed across the genome (Barton and Bengtsson 1986), it would be difficult to maintain neutral differentiation in Apahida since the last ice ages (Vines et al. 2003). In fact, there are reasons to believe that this particular contact zone is much more recent. Variation among transects in genotype and habitat distribution is a common theme in the hybrid zone literature (Harrison and Larson 2016) and likely associated with variation in barrier strength. Nevertheless, the slow diffusion of neutral genetic variants should buffer many hybridizing taxa with extended distribution ranges against local, episodic spates of gene flow.

Under these para‐allopatric conditions (Coyne and Orr 2004, p. 115), there is no antagonism between selection and gene flow. This has two important consequences for the evolution of reproductive isolation. Firstly, any speciation mechanism may contribute (e.g., Kondrashov 2003) and the relative role of ecological divergence remains to be determined in any case. Note that polygenic models of adaptive divergence predict that this role may be limited (Barton 2001; Chevin et al. 2014), unless strongly deleterious epistatic interactions (i.e., Dobzhansky‐Muller incompatibilities) arise in the process (Chevin et al. 2014). Secondly, there are no constraints on the genetic architecture of increasing divergence. In contrast, local adaptation under selection‐gene flow balance between two finite, panmictic populations tends to favor a clustered genomic distribution of selected loci that can gradually evolve in redundant genetic systems (Yeaman and Whitlock 2011). This architecture facilitates the emergence of novel ecotypes, especially when traits under divergent natural selection also cause assortative mating (magic traits, Gavrilets 2004; Servedio et al. 2011), but generates comparatively weak genetic barriers to gene flow in hybrids (Barton and Bengtsson 1986).

The puzzle of continued interbreeding despite profound ecological divergence in *Bombina* illustrates that speciation tends to defy rules (Coyne and Orr 1989) and argues for an open‐minded exploration of the problem. On balance, rapid evolution of reproductive isolation between local ecotypes faces three challenges: a limited gene pool from which to draw mutations that drastically reduce gene flow, the trend toward a clustered genomic distribution of selected traits and the need to build up irreversible reproductive barriers before further environmental change reverses the process. This suggests that many young ecotypes will be ephemeral (Futuyma 1987; Rosenblum et al. 2012). Adaptation to abundant and widely distributed resources provides an alternative path to speciation by bolstering persistence first and by facilitating the evolution of reproductive isolation later. This view affirms the importance of ecology in generating biological diversity: young ecotypes can play important roles in their respective communities and even prompt diversification in other taxa (Abrahamson and Blair 2007), irrespective of whether they are reproductively isolated. We hasten to add that this view has a very long history indeed (Darwin 1859; see Mallet 2008).

Associate Editor: J. Good

Handling Editor: M. Servedio

## Supporting information

Additional Supporting Information may be found in the online version of this article at the publisher's website:

Supporting Information A.
Supporting Information B.
