# Supplementary material for: Para‐allopatry in hybridizing fire‐bellied toads (Bombina bombina and B. variegata): Inference from transcriptome‐wide coalescence analyses
Source: Evolution. 2016 Jul 8;70(8):1803–18. doi: 10.1111/evo.12978 (PMC5129456; doi:10.1111/evo.12978)
Supplement: Supplementary file 1 — Supporting Information A. Supporting Information B. [file EVO-70-1803-s001.zip › evo12978-sup-0001-SuppMat.pdf]

Nürnberger, Lohse, Fijarczyk, Szymura and Blaxter

Para-allopatry in hybridising fire-bellied toads (*Bombina bombina* and *B. variegata*): inference from transcriptome-wide coalescence analyses

Supporting Information A

TABLE OF CONTENTS PAGE

Bioinformatics pipeline 1

1. Defining paralogues 2
2. Analysing pairwise nucleotide alignments for paralog identification 3
3. Peptide and nucleotide alignments within orthogroups 4
4. Why a joint Trinity/Roche 454 assembly was not implemented 5
5. Assessing the paralogue filter (Trinity contigs only) 5
6. SNP filtering 7

Allele specific expression 8

References 9

Bioinformatics pipeline

Overview

As explained in the Methods, a reference transcriptome was defined for each *Bombina* taxon, which included presumptive paralogs. These were identified based on sequence identities in pairwise alignments either within Trinity components or in high scoring BLASTN pairs of Trinity (database) and Roche 454 contigs (query, the top ten hits were analysed). Roche 454 data were only available for *B. bombina* and *B. v. variegata*. Predicted open reading frames within the reference transcriptomes were clustered with OrthoMCL and these clusters were filtered as described in Methods. For pairs of *Bombina* taxa, peptide sequences from robust orthogroups were aligned and back-translated into the nucleotide sequences of the references. Following variant identification and filtering (GATK), an alternate haplotype was constructed for each taxon in a pairwise alignment and added to this alignment. Here, we present in detail the design and logic of this pipeline, explain why we chose not to generate a joint Trinity/Roche 454 assembly for *B. bombina* and *B. v. variegata* and assess the performance of the paralogue filter relative to a commonly used alternative approach.

1. Defining paralogues

As outlined in Methods, paralogues were identified in the first instance based on pairwise alignments with mean sequence identity below 0.98. In cases where there was heterogeneous sequence identity across an alignment, two patterns were distinguished:

(a) an abrupt drop in sequence identity from near 1.0 to an average of about 0.3 that often coincided with the end of exon annotations (ORF prediction and/or BLASTX match to *S. tropicalis*) in one contig only (Fig. A1a). This pattern is consistent with the existence of alternatively spliced terminal exons that cause alignment errors. We exclude such tracts from the computation of overall mean sequence identity.

(b) We found distinct poorly aligned regions in fully annotated sections of otherwise near-perfect alignments, often without any nearby indels. This pattern is consistent with the alternative expression of duplicated exons (Fig. A1b), i.e. the presence of a paralogous tract within a gene.

Because the identification of exons from open reading frame predictions and BLASTX matches is imperfect (*e.g.* due to frame shifts), we devised a heuristic criterion to distinguish these two cases based solely on sequence identity. As shown in Figure A1c, poorly aligned tracts with pattern (a) typically had mean sequence identities below 0.5. Poorly aligned tracts with mean identity above 0.5 matched the distribution of identities in homogeneous, paralogous alignments (Figure A1c, inset). We therefore use a mean identity of 0.5 as the threshold to distinguish between cases (a) and (b) among distinct poorly aligned tract

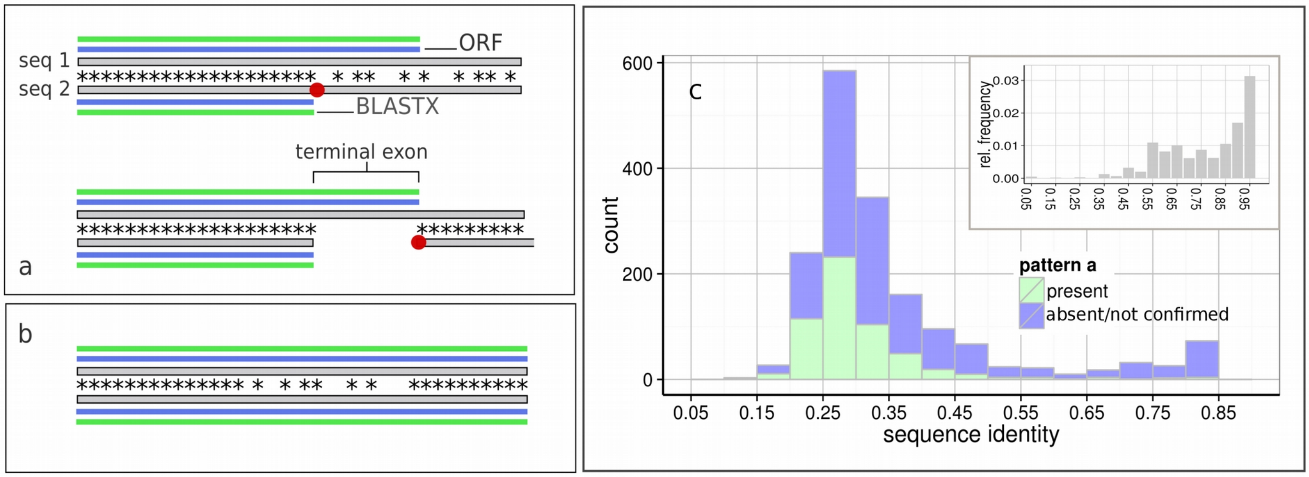

Figure A1. Defining paralogues in the case of heterogeneous sequence identity along alignments. a) Poorly aligned tracts near either the 3' or the 5' end of an alignment that coincide with the end of an exon in one taxon only and suggest the existence of an alternatively spliced terminal exon. Limited sequence information prevents the aligner from opening a gap at the red dot that would bring the tracts back into alignment (bottom sketch in panel a). b) Distinct poorly aligned tracts within an annotated part of the alignment. c) Distribution of mean sequence identity in distinct poorly aligned sequence tracts. The green portions of bars represent alignments in which pattern (a) was identified. The inset plot shows the distribution of homogeneous sequence identities within components. The interval (0.95,1.0] contains 88% of this distribution and has been omitted for clarity. Note that 99.7% of sequence identities between taxa within orthogroups also fall into this interval.

2. Analysing pairwise nucleotide alignments for paralog identification

Pairwise nucleotide alignments were computed with MUSCLE *v.* 3.8.31 (Edgar 2004)

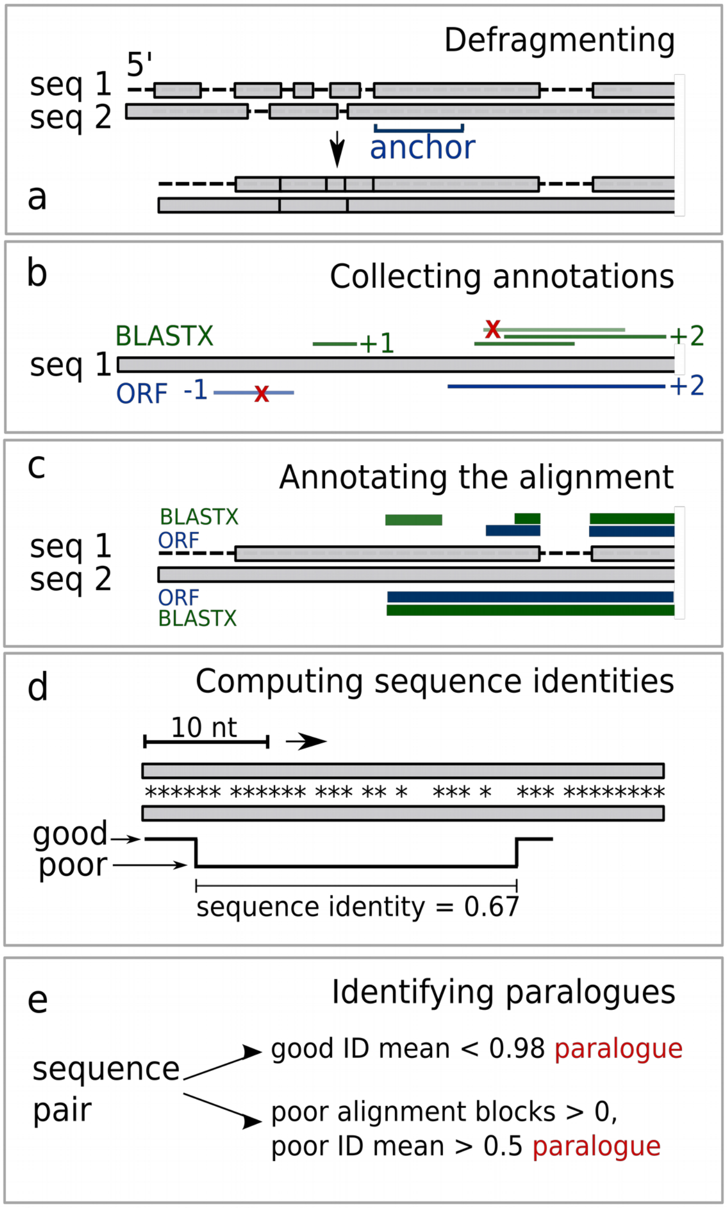

Figure A2. Identification of presumptive paralogues from pairwise sequence alignments. See text for explanation.

Defragmentation of alignments - The 5' and 3' terminal sections of these alignments were often highly fragmented, which presumably reflected true structural variation coupled with limited sequence information near contig ends (Fig. A2a). Without well aligned tracts on either end of an indel, the aligner failed to position gaps correctly. The same behavior was observed in Clustal Omega (Sievers et al. 2014) and Smith-Waterman alignments (EMBOSS water, Rice et al. 2000) and could not be rectified by varying input parameters. We chose to remove implausible, terminal gaps and to treat the resulting low sequence identity in these tracts as an indicator of hidden structural variation (see above). Alignments were scanned for 5' and 3' anchors, *i.e*. the most distally located well aligned tracts, and any gaps outside the anchors were removed. Anchors were defined as either (a) more than 30 bases with sequence identity of at least 0.95 or (b) more than 50 bases with sequence identity of 0.85 or more.

Collecting annotations - Contigs were annotated with BLASTX matches to the *Silurana (Xenopus) tropicalis* proteome (expect-value cutoff = 0.01) and with open reading frame predictions (cf. Methods, Fig. A2b). BLASTX hits were added to the list of annotations one by one starting with the best match. Those hits were discarded that overlapped for more than 50% of their length with previously added ones. All identified ORFs were also added to the list. Per *Bombina* taxon, the number of contigs with more than one ORF prediction ranged from 922 (*B. orientalis*) to 2422 (*B. v. variegata*). Such contigs were found in 4 - 7% of Trinity components (and Roche 454 contigs) with at least one ORF. In those cases in which pairs of contigs had annotations both on the plus strand and the minus strand, the orientation with the larger number of annotated nucleotides was identified. Only BLASTX matches and ORF predictions with that orientation were

retained.

Annotating the alignment - Annotation coordinates of contigs were translated into alignment coordinates (Fig. A2c). For each contig, a set of contiguous sequence blocks was defined, each characterized by its type (unannotated, BLASTX, ORF or BLASTX+ORF), start point and frame. From these data, we recorded indicators of biological features (*i.e.* start and stop codon, total length and position of annotated sequence, alternatively spliced exons, frame shifts) that assisted with the correct computation of sequence identities. For example, the annotation of poorly aligned terminal sequence tracts suggested the occurrence of alternatively spliced terminal exons (see above). ORF predictions with different reading frames in one and the same contig and indels of non-3*n* lengths that were embedded in annotated alignment stretches suggested frameshifts. They were found in 7-9% of Trinity contigs and 30% of Roche 454 contigs. Since they cause especially BLASTX matches to terminate prematurely, they limit the completeness and reliability of annotations. We therefore infer paralogue status based on sequence identity only.

Computing sequence identities - To allow for heterogeneity across an alignment, identities were computed as the proportion of matching positions in ungapped sliding windows (length: 10 nucleotides, Fig. A2d). Identity estimates below 0.9 (*i.e*. two or more mismatches per window) were stored separately, yielding separate mean identities for 'good' and 'poor' alignment stretches. In addition, the total number of nucleotides in each alignment category and the number of separate poor tracts were recorded.

Designating paralogues - Following the rationale laid out above, two criteria were used to identify presumptive paralogues: paralogue pairs had (a) a mean sequence identity in 'good' alignment tracts below 0.98 and/or (b) a mean identity greater than 0.5 in 'poor' alignment tracts, if present (Fig. A2e). If only a single overall mean identity per alignment is computed, distinct low identity tracts that are incompatible with allelic variation at a single locus are missed, especially in long alignments. Condition (b) also assures that alignment errors, which typically result in identities below 0.5, are not taken as evidence of paralogue status (see above).

3. Peptide and nucleotide alignments within orthogoups

The robust orthoset that resulted from OrthoMCL clustering and subsequent filtering steps (cf. Methods) was used to generate cross-taxon sequence alignments for the coalescence analysis. Here we considered only the Illumina data, corresponding to one diploid individual per taxon. We also excluded all orthogroups that contained contigs with more than one ORF prediction. In many cases, these will reflect frameshift errors and thus do not necessarily compromise the analysis: only the correctly predicted peptide sequences would align across taxa and could so be used for inference. However, some of these contigs may contain true overlapping genes. Note that about 7.4% of orthologous human-mouse genes overlap with at least one other gene (Sanna et al. 2008). Thus, nucleotide sequences in some multi-ORF contigs may be subject to complex selective constraints that invalidate the use of fourfold degenerate sites from any one ORF in coalescence analyses.

Per orthogroup, Clustal Omega (Sievers et al. 2014) alignments of predicted peptide sequences were computed for pairs of *Bombina* taxa, including *S. tropicalis* if present. If an orthogroup contained two (or more) peptide sequences from the same taxon (but from separate contigs) and if these sequences did not overlap, these were collapsed into a single cumulative sequence for that taxon. ORFs from fragmented transcript assemblies could thus be jointly analyzed.

Gap-free *Bombina* alignment tracts were identified with Gblocks v. 0.91b (Talavera and Castresana 2007) with default parameters except for the following: minimum aligned sequences at all positions (b1, b2) = 2, maximum number of contiguous non-matching positions (b3) = 4 (default = 8), minimum block length (b4) = 20 (default = 10). For each Gblock and taxon, the corresponding contig name was retrieved and the nucleotide sequence was extracted with PAL2NAL (Suyama et al. 2006). To these cross-taxon alignments, alternate haplotypes for each taxon were then added (see Methods).

The code for the paralogue filter is available at https://github.com/beanurn/orthoset. We note that the code is heavily customized for the specific samples and the assembly software used (particularly Trinity). It would need extensive modification to be used on other datasets.

4. Why a joint Trinity/Roche 454 assembly was not implemented

The pairwise Trinity/Roche 454 alignments of BLASTN matches predicted limited gain in contig lengths from a cross-platform assembly. For the longer of the two aligned contigs (the Trinity contig in ~82% of pairs), the expected gain in length from assembly was no more than 50 bp in 75% of cases. A gain of more than 250 bp would be expected in just under 7% of aligned pairs. While an assembly may join multiple contigs rather than just pairs, preliminary trials (using CAP3) showed that this occurred in a only small fraction of cases (results not shown).

We therefore retained separate contigs of the same gene from two different individuals per taxon rather than risk introducing assembly errors. Roche 454 contigs contributed paralog information to the filtering of orthogroups, but were not part of the coalescence analysis.

5. Assessing the paralogue filter (Trinity contigs only)

In order to illustrate the properties of the paralogue filter (PF), we compare the orthoset that it generates to that obtained by a simpler, commonly used alternative: for each component, only the contig with the longest ORF is retained; after cross-taxon clusters have been generated only those clusters are kept that contain exactly one contig per taxon. We refer to this as the standard approach (STD). Only the Trinity assemblies were used here and separate OrthoMCL analyses were run, each including the *S. tropicalis* proteome. Additional filters were applied as before and equally in both analyses (PF and STD, Fig. A3).

We first consider the representation of contigs in both orthosets. In the case of *B. bombina*, 4776 contigs were present in one or both robust orthosets. The number of contigs that occurred only in one set but not in the other were 240 (STD) and 569 (PF). When combined, these contigs represent 17% of the total (4776). The analogous figures were similar for the other *Bombina* taxa with a maximum of 20% of contigs that featured in only one of the two orthosets.

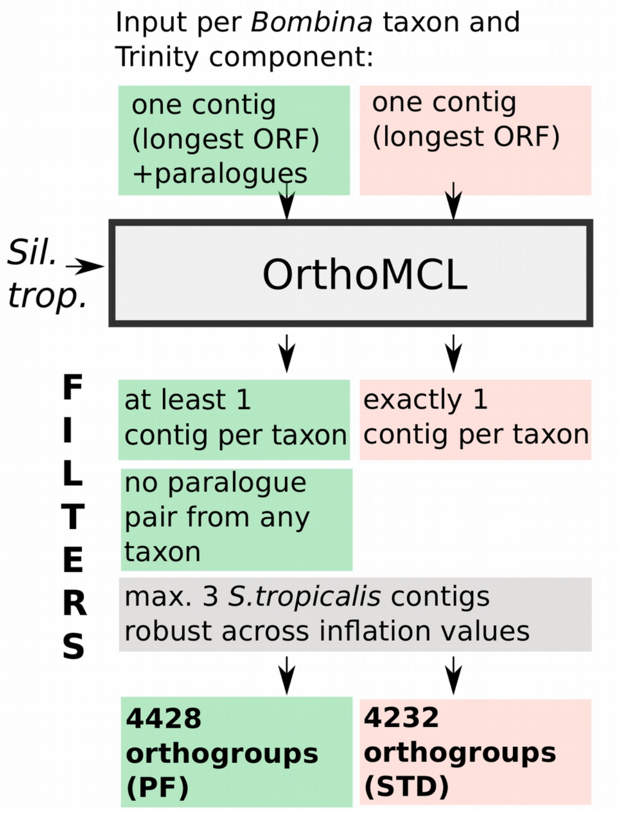

Figure A3. Comparison of paralogue filter (PF) with the simpler 'standard' approach (STD).

Contigs excluded by the PF must have been assigned to clusters that contained paralogue pairs. Because only one contig per component is entered into the OrthoMCL analysis in the STD approach, available information on paralogues is suppressed. Perhaps surprisingly, a relatively larger number of contigs was excluded by the standard analysis but retained by the paralogue filter. This was true for each *Bombina* taxon. This pattern is largely produced by clusters that comprise two (or more) contigs from different components for a given taxon, as would be expected in the case of fragmented gene assemblies. Overall, there were 379 orthogroups that showed this pattern. By allowing such fragmented assemblies to contribute to the orthoset, the PF makes fuller use of the available data. This feature can also account the for the relatively larger number of robust orthogroups produced by the PF (Fig. A3).

The overall effect of the PF on the definition of a robust orthoset across four *Bombina* taxa can be summarised as follows: 716 OrthoMCL clusters with presumptive paralogues were removed, sequence data informed the inclusion of one but not the other of a paralogue pair in 254 orthogroups and, as discussed above, 379 credible orthogroups were retained that would have been removed by a STD analysis. Taken together, these 1349 orthogroups amount to roughly 1/3 of the final set of 4040 groups in the full analysis. While one of these figures (379) is derived from a reduced dataset (excluding the Roche 454 data) and so not strictly comparable, they do jointly illustrate that the paralogue filter makes an important contribution to the definition of a high confidence orthoset

Figure A4 provides an example of improved orthologue assignment in a gene with likely alternate exon use. The assemblies of all four *Bombina* taxa contained a component with high similarity to acid phosphatase 1 in *S. tropicalis* (Figure A4, bottom panel). A paralogue pair was identified within each of these components. OrthoMCL distributed the pairs over two clusters, each containing a mix of 'a' contigs (with longest ORF) and 'b' contigs (middle panel). Peptide alignments (total length: 158 amino acids) showed

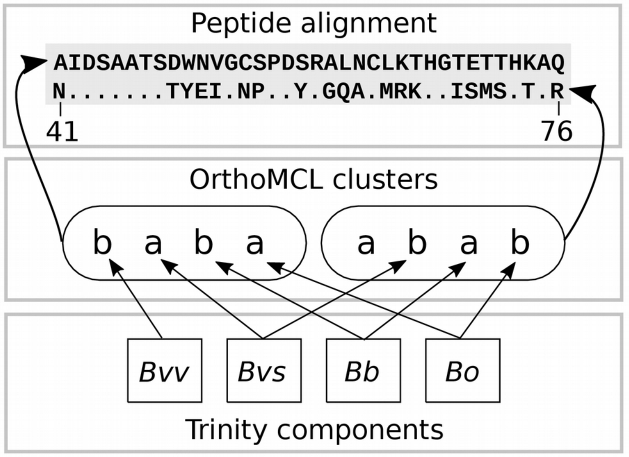

Figure A4. Orthology assignment in a gene with similarity to acid phosphatase 1 in *S. tropicalis*

that the clusters differed only in the middle portion of the protein (position 41 - 76, top panel). All other amino acid positions were identical. The standard approach of including only 'a' contigs in the OrthoMCL analysis resulted in a single cluster that mixed isoforms and so would have led to erroneous estimates of pairwise sequence divergence.

6. SNP Filtering

We based our filtering on the recommendations in the GATK guide 'Calling variants in RNAseq' (https://www.broadinstitute.org/gatk/guide/article?id=3891, last accessed Jan 2015). We applied the GATK VariantFiltration tool to the raw SNP sets, with the following RNA-seq-specific criteria.

```
--filterExpression "QD < 2.0 || FS > 30.0 || MQ < 30.0"  -window 35
-cluster 3
```

where

QD = quality by depth: QUAL / DEPTH (= unfiltered depth)
FS = FisherStrand: assesses uneven representation of a variant among reads from one or the other DNA strand
MQ = mapping quality

Any variant for which the `filterExpression` is true, will be flagged as having failed the RNA-seq filter. Also, any variant in a set of three or more SNPs within a 35 base window will be flagged as belonging to a `SnpCluster`. See the GATK documentation for full explanations.

Table A1 lists the number of SNPs found in blocks of 150 fourfold degenerate sites on which we based our analysis, with separate counts for the SNPs that occured in clusters and those that failed any of the other filtering criteria. In addition to weighing sensitivity versus specificity, we considered biases that might result from excluding some of the data.

Table A1. Properties of SNPs that were detected in blocks of 150 fourfold degenerate sites.

Taxon	#SNP	SnpCluster *	FAIL*	Mean # of SNP per block (PASS)		# SnpClusters with *n* SNP in coalescence dataset		
				no Cluster	Cluster	*n*=1	*n*=2	*n*=3
B. v. variegata	424	25 (5.9)	17 (4.0)	1.70	2.40	19	3	
B. v. scabra	595	40 (6.7)	28 (4.7)	1.51	1.67	22	6	2
B. bombina	253	19 (7.5)	13 (5.1)	1.34	1.60	10	3	1
B. orientalis	1418	116 (8.2)	72 (5.1)	1.81	1.81	57	1	

* count (percentage)

Blocks with SnpClusters tend to have a higher number of other high-quality variants that pass all filters (Tab A1). Since blocks with a large number of heterozygous sites are key indicators of gene flow, we would bias our results if members of SNP clusters were entirely removed. The effect of including them,

however, is moderate, because we sample SNPs intermittently (at fourfold degenerate sites). Most SnpClusters (108/124) added only one SNP to the block in question (Tab. A1).

We therefore retained the members of SnpClusters but removed low quality SNPs (FAIL in Tab. A1) from the dataset. In retrospect, the results of the coalescence analysis validate the inclusion of SNPs from clusters. In both cases of inferred post-divergence gene flow, the direction was into the *B. v. variegata* gene pool. An undue inflation of SNP counts per block due to SnpClusters is least likely in this taxon (Tab. A1).

Allele specific expression

Figure A5 displays the distributions of the major allele frequency per SNP in the contigs that supplied blocks of 150 fourfold degenerate sites to the coalescence analysis. Data are from the taxa with the lowest (*B. orientalis*) and the highest (*B. v. variegata*) median read depth, respectively. Panels show the data for different read depth categories (numbers in the grey vertical bars are upper bounds). The count (and proportion) of SNPs is listed inside each panel.

We have not conducted rigorous tests of allele specific expression. The red bars under the x-axes show the critical values in major allele frequency for a simple binomial test (Ho: p = 0.5) that correspond to an α-level of 0.01. The left and right edges of each bar apply to highest and lowest read depth in each panel, respectively. Clearly, there are numerous SNPs in each taxon for which a single binomial test would reject the null hypothesis of equal expression.

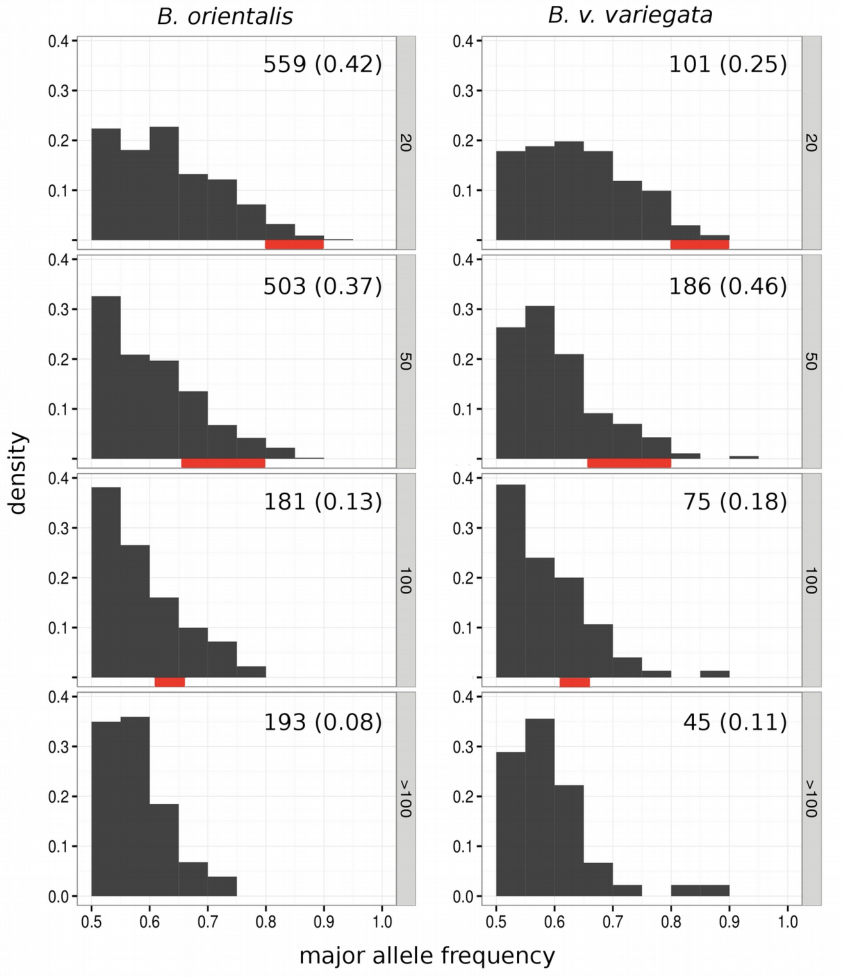

Figure A5. Major allele frequencies for SNPs for two Bombina taxa. Per taxon, allele frequencies were computed across reads coveringa single SNP in a single individual. See text for further explanation.

References

Edgar, R. C. 2004. MUSCLE: multiple sequence alignment with high accuracy and high throughput. Nucleic Acids Res. 32:1792–1797.

Rice, P., I. Longden, and A. Bleasby. 2000. EMBOSS: The European Molecular Biology Open Software Suite. Trends Genet. 16:276–277.

Sanna, C.R., W.-H. Li, and L. Zhang. 2008. Overlapping genes in the human and mouse genomes. BMC Genomics 9: 169.

Sievers, F. et al. 2014. Fast, scalable generation of high-quality protein multiple sequence alignments using Clustal Omega. Mol. Syst. Biol. 7:539–539.

Suyama, M., D. Torrents, and P. Bork. 2006. PAL2NAL: robust conversion of protein sequence alignments into the corresponding codon alignments. Nucleic Acids Res. 34:W609–W612.

Talavera G, Castresana J. 2007. Improvement of phylogenies after removing divergent and ambiguously aligned blocks from protein sequence alignments. Systematic Biology 56: 564-577.
